# Effects of repetitive exercise and thermal stress on human cognitive processing

**DOI:** 10.14814/phy2.14003

**Published:** 2019-02-21

**Authors:** Manabu Shibasaki, Mari Namba, Yoshi‐Ichiro Kamijo, Tomoyuki Ito, Ryusuke Kakigi, Hiroki Nakata

**Affiliations:** ^1^ Faculty of Human Life and Environment Department of Health Sciences Nara Women's University Nara Japan; ^2^ Graduate School of Humanities and Sciences Nara Women's University Nara Japan; ^3^ Department of Rehabilitation Medicine Wakamaya Medical University Wakayama Japan; ^4^ Department of Rehabilitation Medicine Kyoto Prefectural University of Medicine Kyoto Japan; ^5^ Department of Integrative Physiology National Institute for Physiological Sciences Okazaki Japan

**Keywords:** Aerobic exercise, event‐related potentials (ERPs), internal temperature, repetitive exercise

## Abstract

Cognitive performances may improve after acute moderate exercise, but not after prolonged and/or heavy exercise. The present study aimed to investigate the effects of environmental temperature during exercise on human cognitive processing. Fifteen healthy males performed four bouts of a 15‐min cycling exercise with a 10‐min rest between each bout, and event‐related potentials (ERPs) were recorded in five sessions during somatosensory Go/No‐go paradigms (i.e., Pre, post‐first exercise bout, post‐second exercise bout, post‐third exercise bout, and post‐fourth exercise bout) in an environmental chamber with temperature controlled at 20°C (Temperate) and 35°C (Hot). Increases in external canal temperature and heart rate were greater under the 35°C condition than under the 20°C condition. Regardless of thermal conditions, reaction times (RT) and error rates were not affected by the repetition of moderate exercise, whereas the peak amplitude of the N140 component, which is mainly related to somatosensory processing, was significantly reduced with the repetition of the exercise. However the peak amplitude of P300, which is linked to cognitive processes of context updating, context closure, and event‐categorization, was significantly smaller in post‐third and post‐fourth exercise bouts under the 35°C condition than under the 20°C condition, and this decrease was more prominent in No‐go trials under the 35°C condition. These results suggest that executive function, which is based on RTs and error rates, is not affected by prolonged exercise and different thermal conditions, whereas the exercise in a hot environment impairs human cognitive processing, particularly response inhibition.

## Introduction

Exercise training or habitual physical activities elicit favorable changes in structural and functional brain networks (Gomez‐Pinilla and Hillman 2013; Mandolesi et al. 2018). The effects of sustained exercise on brain function as well as morphological changes have been objectively demonstrated by magnetic resonance imaging (MRI) studies (Gomez‐Pinilla and Hillman 2013; Park and Friston 2013; Mandolesi et al. 2018). The acute effect of exercise trials is thought to improve brain function, but equipments and indices are limited to obtain physiological evidence because the changes induced by acute exercise may be transient. The most convenient and flexible equipment for evaluating brain function, electroencephalography (EEG), or near‐infrared spectroscopy may be suitable for such research. Attention or orientation as a cognitive function index during or after exercise can be evaluated by an EEG power spectrum obtained from a frequency analysis or event‐related potentials (ERPs) by time‐locked averaging EEG (Kamijo et al. 2004; Rasmussen et al. 2004; Gomez‐Pinilla and Hillman 2013). However, because the modality, intensity, frequency, duration, and combination of these are markedly variable, the effects of exercise on cognitive function remain unclear.

EEG‐ERPs are widely used to evaluate the reception and processing of sensory information, as well as higher level processing that involves selective attention, memory updating, semantic comprehension, and other types of cognitive activities (Duncan et al. 2009). Exercise intensity appears to influence cognitive performance in an inverted‐U effect (Brisswalter et al. 2002; Kamijo et al. 2004; McMorris and Hale 2012). Using EEG‐ERPs, acute moderate exercise exerts positive effects on cognitive function, whereas light and heavy exercise do not (Kamijo et al. 2004, 2007), however, this effectiveness appears to be dependent on duration of exercise (Ludyga et al. 2016). A relatively short period (~30 min) of moderate exercise was shown to be beneficial for cognitive function, whereas prolonged exercise at not only moderate, but also low and high intensities did not induce improvements (Kamijo et al. 2004, 2007; Grego et al. 2005; Loprinzi and Kane 2015; Olson et al. 2016). Exercise‐induced central and/or peripheral fatigue and/or increases in body temperatures due to exercise may contribute to this ineffectiveness. A markedly increased internal temperature is a limiting factor during exercise in the heat because cardiovascular and neuromuscular responses decrease during exercise in the heat (Gonzalez‐Alonso et al. 1999; Nybo and Nielsen 2001; Simmons et al. 2008). We recently demonstrated that increases in internal temperature during passive heat stress decreased cognitive function (Shibasaki et al. 2016, 2017). Therefore, we hypothesized that an increase in body temperature during exercise contributes to the modulation of cognitive function. In order to investigate the effects of increases in body temperature on exercise‐related changes in cognitive function, we designed the present study to consist of four bouts of a 15‐min moderate cycling exercise in temperate and hot environments.

## Methods

### Subjects

Fifteen male subjects participated in the present study. The age, body mass, and height of the subjects were 20.8 ± 0.9 years, 83.6 ± 8.1 kg, and 173.2 ± 6.5 cm. Participants were college rugby players and baseball players who were trained 4–5 days per week. No subjects had a history of adverse medical conditions, including neurological or psychiatric disorders. All subjects were informed of the study protocol and risks before providing their written informed consent. The present study was approved by the Ethics Committee of the Nara Women's University, Nara, Japan. The protocol was performed in accordance with the Declaration of Helsinki.

Subjects performed four bouts of an interval cycle exercise in a temperature‐controlled environmental chamber (TABAI ESPEC, Osaka, Japan), which was set to two different conditions (20°C and 35°C, relative humidity was regulated between 30 and 40%). They performed the same absolute workload under both conditions. There was at least a 3‐day interval between the 20°C and 35°C conditions, and experiments were performed at the same time on a different day in a random order. On the first visit, exercise intensity was assessed in the anteroom under 20°C (not in the environmental chamber). Subjects wearing an electrocardiogram (ECG) monitoring device (Biomulti 1000, NEC, Tokyo, Japan) sat on a cycle ergometer (Ergomedic 874E, Monark, Sweden) in order to evaluate their workload. Exercise intensity was set to maintain a heart rate (HR) of approximately 130 bpm (~67% HR max) for 15 min of cycling in a temperate environment. The time interval between each bout of exercise was set at 10 min. A metronome sound was presented (Dr. Beat DB‐66, Boss, Shizuoka, Japan) to maintain smooth constant pedaling at 60 rpm. Exercise intensity was kept constant throughout the experiments under both conditions (132 ± 27 W). Subjects did not drink water throughout the experiment.

On their arrival at the laboratory, subjects weighed themselves nude on a scale (A&D HW‐100KGL, 10 g accuracy) with an empty bladder, and then wore only underwear and shorts. Subjects walked into the environmental chamber and rested quietly on a comfortable armchair for ~30 min. During the resting period, the following measurement equipment was attached. External canal temperature (Tear) was continuously measured using an infrared sensor (Nipro CE Thermo, NIPRO, Osaka, Japan) in the left ear canal, and sampled at 20 Hz via a data acquisition system (MP150, BIOPAC Systems, Santa Barbara, CA, USA). We previously confirmed that temperature measured using this device was reliable as an index of internal temperature (Ogoh et al. 2013; Shibasaki et al. 2017). Skin temperatures were measured using copper‐constantan thermocouples at six sites (the chest, abdomen, upper and lower back, thigh, and calf), and stored at 1‐sec intervals using another data acquisition system (DA100, YOKOGAWA, Tokyo, Japan). Mean skin temperature (Tsk) was calculated from the weighted average of six points (Taylor et al. 1989). HR was measured continuously using ECG (Biomulti 1000, NEC, Tokyo, Japan). Arterial blood pressure was measured before and at the end of the exercise by auscultation of the brachial artery via electrosphygmomanometry (STBP‐780, Colin, Tokyo, Japan). EEGs were recorded with Ag/AgCl disk electrodes placed on the scalp at Fz, Cz, Pz, C3, and C4, and all EEG signals were collected on a signal processor (Neuropack MEB‐2200 system, Nihon‐Kohden, Tokyo, Japan). Each disk electrode placement was marked with a permanent marker. Disk electrodes were removed and replaced in each session to avoid the possible artifact of the sweat rate.

Following instrumentation, subjects received instructions regarding the Go/No‐go task. In the somatosensory Go/No‐go paradigm, the Go stimulus was delivered to the second digit of the left hand, and the No‐go stimulus to the fifth digit of the left hand. Subjects had to respond to the stimulus by pushing a button with their right thumb (contralateral to the stimulated side) as quickly as possible only after the presentation of a Go stimulus. During recordings, subjects were instructed to keep their eyes open and look at a small fixation point positioned in front of them at a distance of approximately 1 m. One run comprised 80 epochs of a stimulation, which included 40 epochs for Go stimuli and 40 for No‐go stimuli. ERPs were recorded in five sessions during the somatosensory Go/No‐go paradigms (i.e., Pre, post‐first exercise bout, post‐second exercise bout, post‐third exercise bout, and post‐fourth exercise bout) under 20°C (temperate) and 35°C (hot) conditions. EEG data were monitored but analyzed after the end of experiment. The practice session consisted of 10 stimuli before the recordings of the Pre session in each visit.

### Data and statistical analyses

Each scalp electrode for EEG was referenced to linked earlobes. The ground electrode was placed at Fpz. Impedance was maintained at less than 5 kohm. The bandpass filter was 0.1–50 Hz. An electrooculogram (EOG) was also recorded to eliminate eye movements or blinks exceeding 100 *μ*V. The analysis epoch for ERPs was 600 msec, including a prestimulus baseline period of 60 msec. Data were collected on a computer with a sampling rate of 1000 Hz. The peak amplitudes and latencies of N140 and P300 were measured at 110–210 and 240–500 msec, respectively. Amplitudes were measured baseline‐to‐peak. Reaction times (RT) were recorded on a computer, and slow responses exceeding 700 msec and incorrect responses were eliminated from averaging. N140 and P300 EEG values were obtained from the averaged responses of the Fz, Cz, Pz, C3, and C4 locations. The mean peak amplitude and latency for these variables, averaged across the five electrodes, were statistically evaluated via a three‐way ANOVA with repeated measures using within‐subjects factors of Condition (20°C vs. 35°C), Session (Pre, post‐ex1, post‐ex2, post‐ex3, and post‐ex4), and Stimulus (Go vs. No‐go). Behavioral data on the mean RT, standard deviation (SD) of RT, and error rate including commission (i.e., response in the No‐go trial) and omission (i.e., no response or a slow response in the Go trial) errors were subjected to repeated measures ANOVAs with Condition and Session as factors. When a significant effect of Condition was observed, the post‐hoc paired *t*‐test was adjusted to identify differences between the 20°C and 35°C conditions. When a significant effect of Session was observed, the post‐hoc paired *t*‐test was adjusted to identify specific differences between the pre and other sessions.

**Figure 1:**
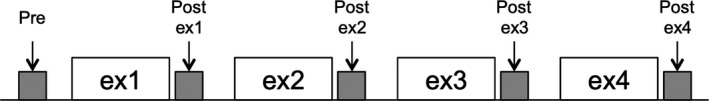
A schematic illustration of the protocol. Somatosensory Go/No‐go paradigms (gray box) performed before first exercise (Pre) and immediately after each exercise bout (ex‐1, ex‐2, ex‐3, and ex‐4).

A 60‐sec average was calculated for temperature variables (i.e., Tear and Tsk) and HR before (i.e., pre) and at the end of each exercise (e.g., ex1, ex2, ex3, and ex4). Unfortunately, the Tear data logger did not record in three of fifteen subjects under either condition. Furthermore, the Tsk data logger did not record in two subjects. Since blood pressure was not measured at the end of exercise in some subjects, data obtained at rest and before each exercise were analyzed. These variables and mean blood pressure obtained before and at the end of each exercise were analyzed by a two‐way ANOVA with repeated measures using the within‐subject factors of Condition and Session. When the significant effects of Condition‐Session were identified, the post‐hoc Student–Newman–Keuls test was adjusted to identify specific differences (Figure 1).

A Mauchly's sphericity assumption evaluation was performed for all repeated measures factors with more than two levels. A Greenhouse‐Geisser adjustment was used if the results of that test were significant. Statistical analyses were conducted using SPSS (Ver. 22). Significance was set at *P* < 0.05.

## Results

**Figure 2:**
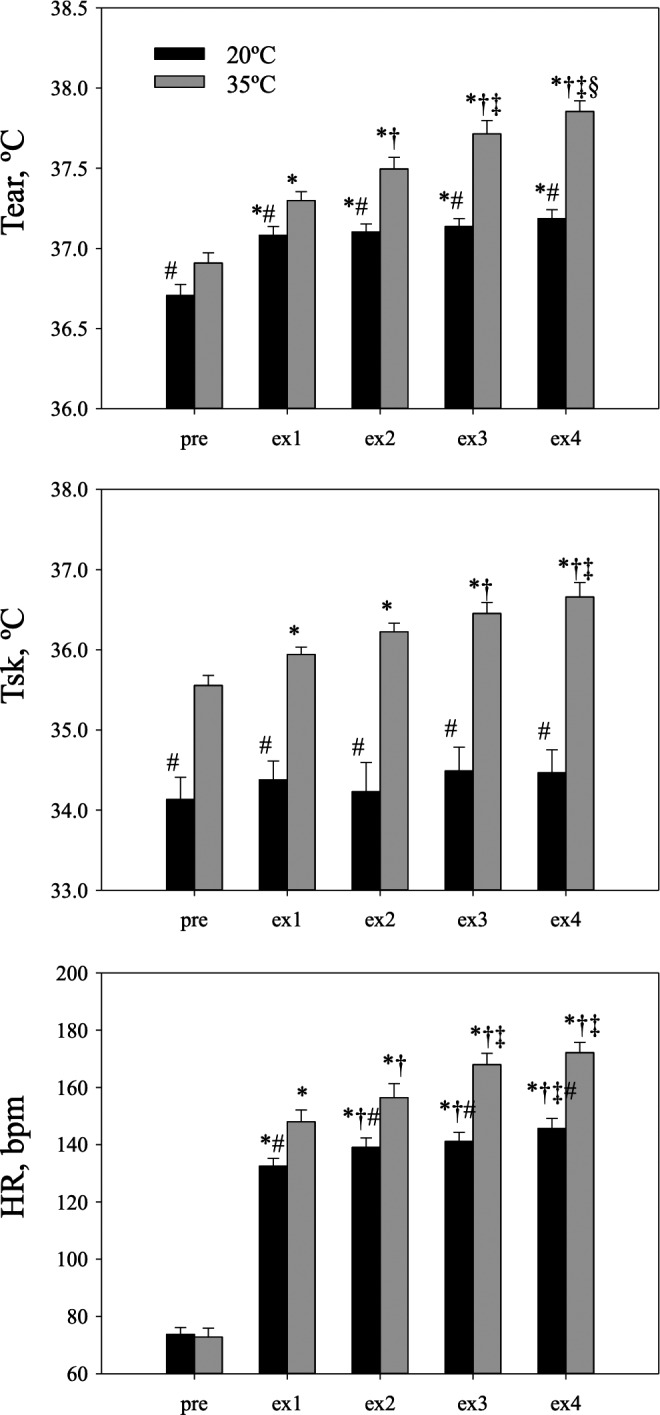
External canal temperature (Tear), mean skin temperature (Tsk), and heart rate (HR) before (Pre) and at the end of each exercise bout (ex‐1, ex‐2, ex‐3, and ex‐4) under the 20°C (temperate, black bar) and 35°C (hot, gray bar) conditions. Bars indicate standard errors. A significant Condition‐Session interaction was observed in each variable (all, *P* < 0.05). *From the Pre, ^†^from Ex‐1, ^‡^from Ex‐2, ^§^from Ex‐3, ^#^20°C versus 35°C.

Figure 2 shows body temperatures and HR. ANOVAs revealed that Tear was significantly higher in the 35°C condition (Condition, Session, and a Condition‐Session interaction; all *P* < 0.001). ANOVAs revealed that Tsk was also significantly higher in the 35°C condition (Condition; *P* < 0.01, Session and a Condition‐Session interaction; both *P* < 0.05). ANOVAs revealed that HR was significantly higher in the 35°C condition (Condition, Session, and a Condition‐Session interaction; all *P* < 0.001). The results of the post‐hoc test are shown in Figure 2. Mean arterial blood pressure was maintained and no significant main effects were observed. Body weight losses under the 20°C and 35°C conditions were 0.96 ± 0.33 and 1.74 ± 0.49 kg, respectively, and the difference was significant (*P* < 0.01).

**Figure 3:**
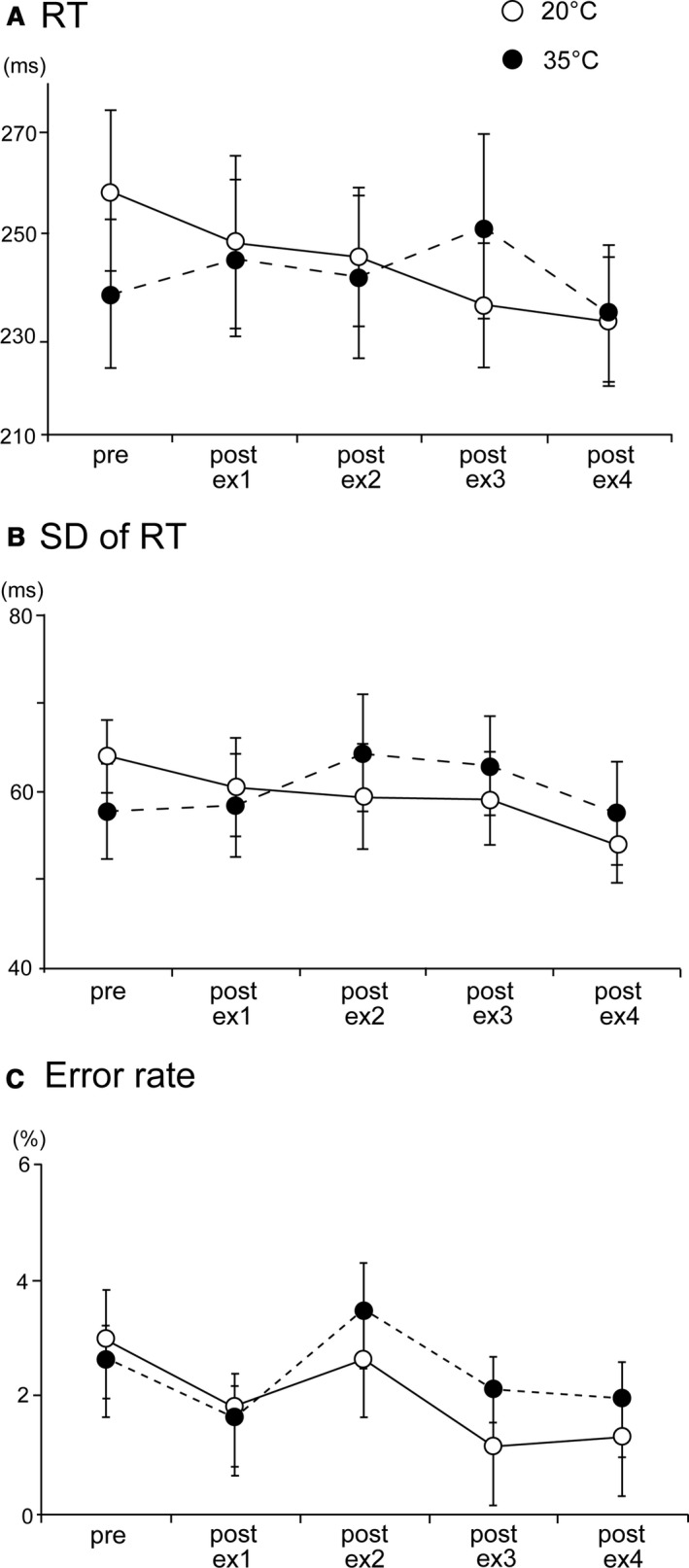
(A) Mean reaction times (RT) for 20°C and 35°C conditions. White circles indicate RT in 20°C, and black circles show RT in 35°C. Bars indicate standard errors. A significant Condition‐Session interaction was observed. (B) The mean standard deviation (SD) of RT for the 20°C and 35°C conditions. (C) The mean error rate for the 20°C and 35°C conditions. ^#^ *P* < 0.05 20°C versus 35°C.

Figure 3A shows mean RT with standard errors (SE). A significant Condition‐Session interaction was observed for RT (*P* < 0.05). This interaction revealed a difference in the mean RT between the 20°C and 35°C conditions with repeated sessions. Further analyses showed no significant main effects under the 20°C and 35°C conditions. Figure 3B shows the SD of RT with SE. No significant main effects or interactions on the SD of RT were observed. Figure 3C shows the error rate with SE. No significant main effects or interactions were noted.

**Figure 4:**
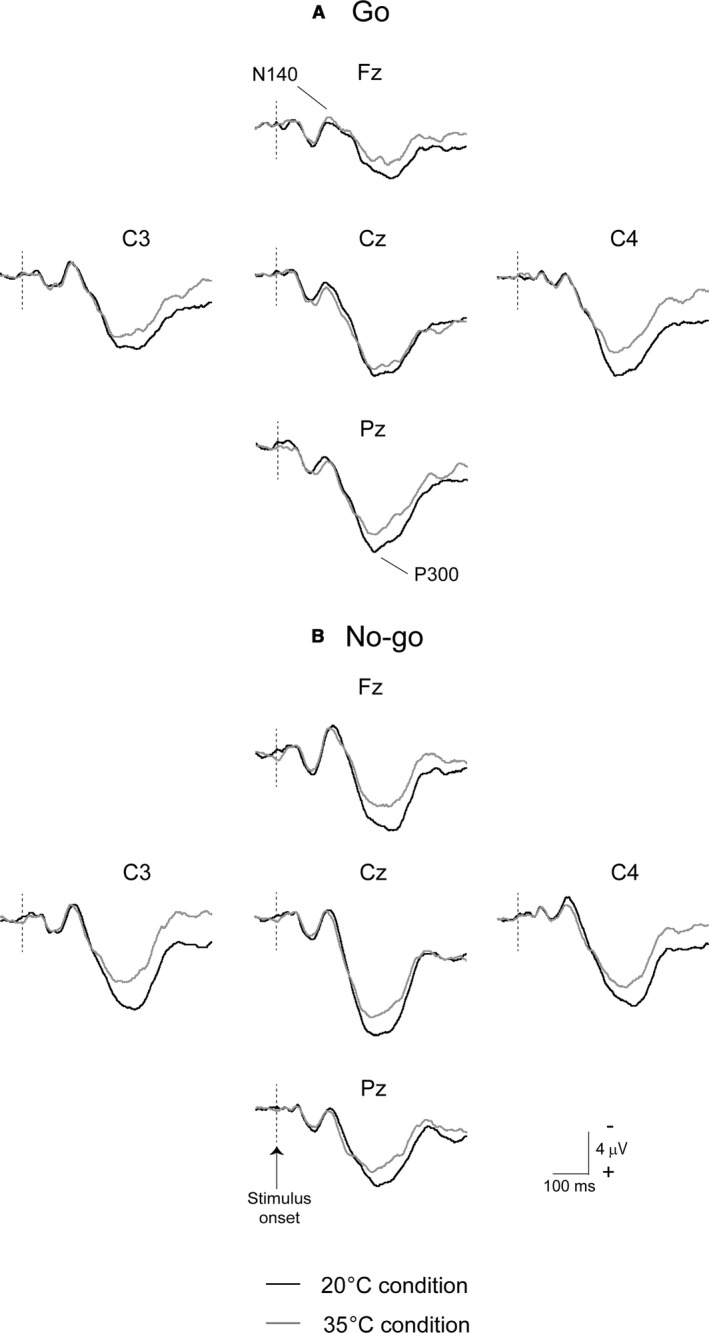
Typical grand‐averaged somatosensory ERP waveforms evoked by Go and No‐go stimuli for 20°C and 35°C conditions in the fourth session. ERP, event‐related potential; RT, reaction times.

**Figure 5:**
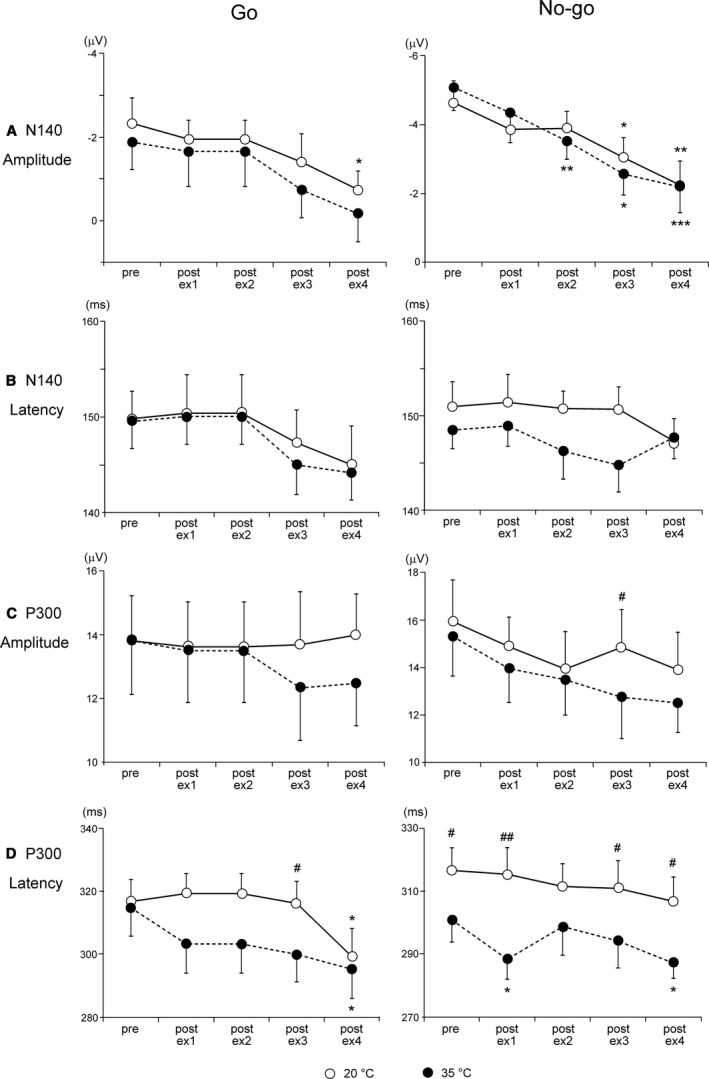
(A) The mean amplitude of N140. Vertical lines indicate SE. (B) Mean latency of N140. (C) The mean amplitude of P300. (D) The mean latency of P300. Data were collapsed across Fz, Cz, Pz, C3, and C4. *P < 0.05 versus first Session; **P < 0.01 versus first Session; ^#^ *P* < 0.05 20°C versus 35°C; ^##^ *P* < 0.05 20°C versus 35°C.

Figure 4 shows grand‐averaged somatosensory ERP waveforms evoked by (A) Go and (B) No‐go stimuli in the fourth session across all subjects (*n* = 15). Black lines indicate waveforms under the 20°C condition, and gray lines show those under the 35°C condition. Figure 5 shows a comparison of conditions and sessions in ERP variables. The peak amplitude of N140 was significantly decreased with both stimuli (Go trials and No‐go trials) and conditions (20°C and 35°C), whereas the peak latency of N140 revealed no significant main effects or interactions. ANOVAs for the peak amplitude of N140 revealed the significant main effects of Session and Stimulus (both, *P* < 0.001). Post‐hoc testing for Session showed that the amplitude of Go‐N140 was significantly larger in pre than in the post‐ex4 session (*P* < 0.01) under the 20°C condition. The amplitude of No‐go‐N140 was significantly larger in the first than in the post‐ex3 (*P* < 0.05) and post‐ex4 sessions (*P* < 0.01) under the 20°C condition, and in the pre than in the post‐ex2 (*P* < 0.01), post‐ex3 (*P* < 0.05), and post‐ex4 sessions (*P* < 0.001) under the 35°C condition. These results suggested that the amplitude of N140 decreased with exercise repetition.

The peak amplitude of P300 was significantly lower in the 35°C condition (*P* < 0.05). The post‐hoc test for Condition in each Session showed that the peak amplitude of No‐go‐P300 was significantly smaller under the 35°C condition than under the 20°C condition in the fourth session (*P* < 0.05). ANOVAs for the peak latency of P300 revealed the significant main effects of Condition and Session (both, *P* < 0.001). The post‐hoc test for Condition in each Session showed that the peak latency of Go‐P300 was significantly earlier under the 35°C condition than under the 20°C condition in the post‐ex3 session (*P* < 0.05). The post‐hoc test for Condition on each Session showed that the peak latency of No‐go‐P300 was significantly earlier under the 35°C condition than under the 20°C condition in the pre (*P* < 0.05), post‐ex1 (*P* < 0.01), post‐ex3 (*P* < 0.05), and post‐ex4 sessions (*P* < 0.05) (Fig. 5D). Since body temperatures were significantly higher in pre, it may affect this difference. Moreover, post‐hoc testing for Session showed that the latency of Go‐P300 was significantly later in the first than in the fifth session under the 20°C and 35°C conditions (*P* < 0.05, respectively). The latency of No‐go‐P300 was significantly later in the pre than in the post‐ex1 (*P* < 0.05) and post‐ex4 sessions (*P* < 0.05) during the 35°C condition (Fig. 5D). These results suggested that the latency of P300 was earlier with the repetition of exercise.

## Discussion

The neural activity of response execution (i.e., amplitude of the Go‐P300 component) as well as RTs did not change in any sessions after each moderate exercise trial under the 20°C (temperate) condition. However, the neural activity of response inhibition (i.e., amplitude of the No‐go‐P300 component) was gradually decreased under the 35°C (hot) condition, and was lower in the hot condition than in the temperate condition. Although the positive effects of acute exercise on cognitive processing were not observed in the temperate condition, the effects of repetitive exercises on cognitive function were observed when internal temperature markedly increased, particularly with the No‐go stimulus in the hot condition.

Exercise increases circulating metabolites and hormones, which may enhance not only physiological function, but also psychological function. For example, catecholamines and brain‐derived neurotrophic factor (BDNF) have been implicated in cognitive performance (Hillman et al. 2003; Kamijo et al. 2004; Chang et al. 2012, 2017). From these findings, coupled with an inverted‐U effect on cognitive performance related to exercise intensity (Brisswalter et al. 2002; Kamijo et al. 2004; McMorris and Hale 2012), the effects of exercise‐related factors on cognitive performance may be due to the interactions among those metabolites and hormones. These substances may be modulated not only by exercise intensity, but also by the duration of exercise. Tsukamoto et al. designed several conditions of exercise (intensity, duration) and demonstrated that moderate exercise intensity and duration improved behavioral executive function (Tsukamoto et al. 2016, 2017). The accumulation of exercise‐induced substances during prolonged or repetitive exercises may gradually impair cognitive performance. Prolonged or repetitive exercise also increases body temperature, resulting in cardiovascular drift and central fatigue (Gonzalez‐Alonso 2007; Nybo et al. 2014). In the present study, we designed the four bouts of exercise protocol to demonstrate the effects of exercise on cognitive processing at two different ambient temperatures. Tear increased under both conditions, but was greater under the 35°C condition than under the 20°C condition. Under the 20°C condition, HR increased to 132 ± 11 bpm at the end of the first bout of exercise, whereas cardiovascular drift was small (HR was 146 ± 14 bpm at the end of the fourth bout). At the same workload in the hot environment, however, HR at the end of each exercise gradually increased and clearly showed cardiovascular drift (Fig. 2).

Nakata et al. (2015a) previously demonstrated that task repetition also affected the amplitudes of N140 and P300 in a somatosensory Go/No‐go paradigm. In that study, subjects performed seven trials with a 5‐min interval between each trial, and both amplitudes decreased with task repetition, particularly in the latter half. Therefore, we carefully designed the present protocol to minimize the effects of task repetition on EEG‐ERPs. Under the 20°C condition, neither executive and inhibitory cognitive processing (i.e., amplitude of the Go‐P300 and No‐go‐P300 components, respectively) nor behavioral responses (i.e., RTs and error rates) changed throughout the four sessions of moderate exercise, whereas somatosensory processing (i.e., amplitudes of the N140 component) was reduced regardless of the stimulus. These results indicate that the generators for Go‐N140 and No‐go‐N140 from several regions, including the secondary somatosensory cortex, insula, cingulate cortex, and medial temporal area, may be less restorable (Nakata et al. 2015a,2015b). Therefore, it may not be an inappropriate protocol for investigating the effects of repetitive exercise on somatosensory processing. On the other hand, we observed clearly different responses in cognitive processing.

**Figure 6:**
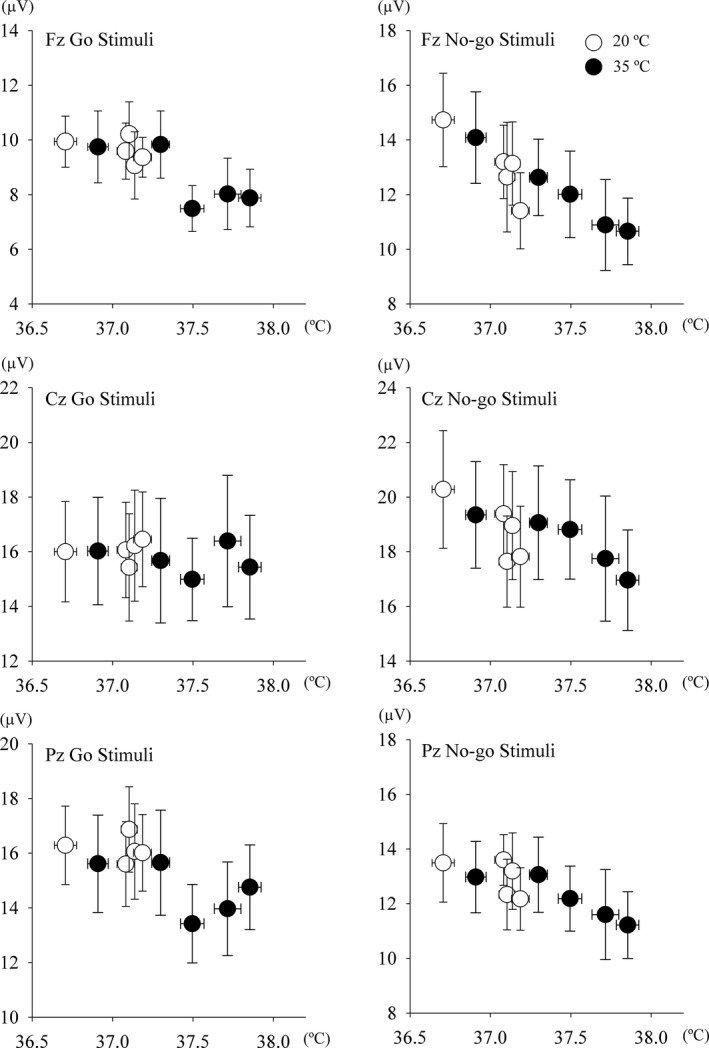
Relationship between external canal temperature (Tear) and the amplitude of N140 during Go stimuli (left) and No‐go stimuli (right) at Fz (top), Cz (middle), and Pz (bottom).

**Figure 7:**
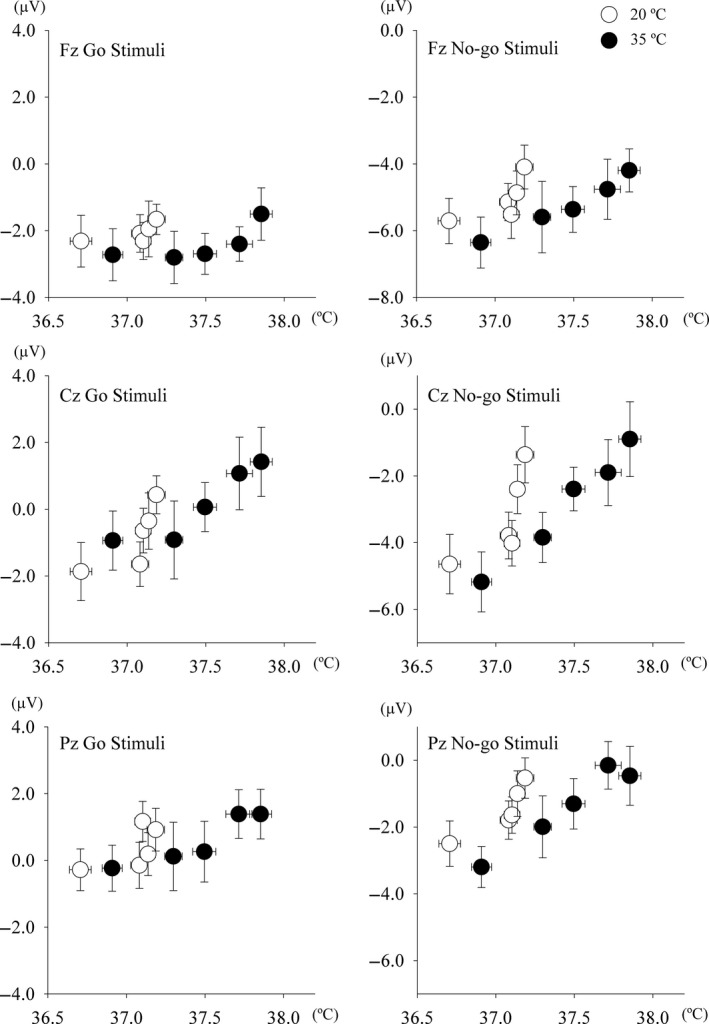
Relationship between external canal temperature (Tear) and the amplitude of P300 during Go stimuli (left) and No‐go stimuli (right) at Fz (top), Cz (middle), and Pz (bottom).

In temporal coincidence to stimuli, voltage changes were observed approximately 300 msec after the stimulus. Stimulation to the oddball test, target stimuli (rare frequent) with the pressing of a button or counting events induced larger amplitudes at the parietal midline (Pz), but none or small amplitudes in standard stimuli. We previously reported greater reductions in the amplitudes of both P300 components under hyperthermic conditions and this impairment lasted longer in the No‐go‐P300 component than in the Go P300 component (Shibasaki et al. 2017). The Go/No‐go paradigms with an even frequency (50% Go stimuli and 50% No‐Go stimuli) used in the present study may show the positive voltage changes with both stimuli and reduce motor‐related activation (Kopp et al. 1996; Verleger et al. 2006). No‐go‐P300 has been interpreted as indicating inhibition, response conflict, or attention‐modulated monitoring (Falkenstein et al. 1999; Fallgatter and Strik 1999; Polich 2007). Moreover, the amplitudes of No‐go‐P300 were elicited at the fronto‐central electrodes (Fz and Cz), and were larger than in the Go trials (Nakata et al. 2015a). Yanagisawa et al. also suggested that improved cognitive behavioral performance after acute moderate exercise may be associated with increased dorsolateral prefrontal cortex activity (Yanagisawa et al. 2010). We expected an increased amplitude in Go‐P300 after the first exercise bout, but decreased or maintained amplitudes in the latter half under the 20°C (temperate) condition. However, the amplitudes of Go‐P300 did not change throughout all sessions in the temperate condition. On the other hand, in the hot condition, we observed different responses between the Go‐P300 and No‐go‐P300 components. Figures 6 and 7 plotted the relationship between internal temperature and the amplitudes of N140 and P300, respectively. The decreased amplitudes of both N140 components were independent of changes in internal temperature (Fig. 6), whereas those of No‐go‐P300 decreased with an increase in internal temperature and those of Go‐P300 did not change (Fig. 7), which is consistent with our previous findings (Shibasaki et al. 2016, 2017).

The neural network associated with executive and inhibitory processing has been examined using *f*MRI, and these processes include the dorsolateral (DLPFC) and ventrolateral prefrontal cortices (VLPFC), supplementary motor area (SMA), primary sensorimotor area (SMI), anterior cingulate cortex (ACC), temporoparietal junction, temporal and parietal lobes, and thalamus (Casey et al. 1997; Nakata et al. 2008a,2008b). Based upon these findings, coupled with the present results and our previous study (Shibasaki et al. 2017), increased internal temperature may reduce neural activities in these regions. Nakata et al. (2008b) previously showed that the strength of neural activity was greater in No‐go trials than in Go trials at the DLPFC, VLPFC, ACC, inferior parietal lobule, and caudate. Although speculative, these areas related to inhibitory function may be impaired by an elevated internal temperature, as shown in Figure 5 right. Alternatively, the neural network of cognitive processing may have become obstructed when brain temperature increased over a given level. Nakata et al. (2015a) also investigated the temporal dynamics of neural activation for somatosensory Go/No‐go trials, and found that activated regions and timing in the primary somatosensory cortex, secondary somatosensory cortex, and SMA were similar between the Go and No‐go trials.

The results in the present study showed that repeated and prolonged exercise may exert stronger effect on inhibitory function. Figure 6 shows the relationship between internal temperature and the amplitudes of Go P300 (left) and No‐go‐P300 (right) at the three regions. A negative relationship was observed between internal temperature and the amplitudes of No‐go‐P300 regardless of regions. However, in addition to elevations in internal temperature, other exercise‐related factor(s) must contribute to this reduction. We previously reported that mild hyperthermia (an ~0.8°C increase in internal temperature) slightly decreased the amplitude of the P300 component (Shibasaki et al. 2016), whereas internal temperature was elevated by 0.91 ± 0.28°C at the end of fourth exercise trial under the 35°C (hot) condition in the present study. Although the increase in internal temperature was not significant, cardiovascular drift was observed in the hot condition. Mean blood pressure at rest (i.e., before exercise) was maintained, whereas body weight loss was greater under the 35°C condition than under the 20°C condition. Although speculative, dehydration may contribute to the reduced amplitude of the P300 components.

Behavioral indices of executive function, such as RTs and error rates, are simple and convenient; however, these did not differ between ambient temperature conditions. Since exercise‐induced benefits on executive function result from improvements in multiple processes including the speed of stimulus classification, stimulus evaluation, response selection, and motor preparation (Doucet and Stelmack 1999), studies combining behavioral and neurophysiological measures allow for further examinations of the underlying mechanisms for RTs and accuracy (Ludyga et al. 2016). However, core cognitive processes include inhibition, working memory, and cognitive flexibility (Gomez‐Pinilla and Hillman 2013). Although the evaluation of EEG‐ERPs is time consuming with many restrictions, the results of the present study showed its potential to clarify the effectiveness of exercise.

An infrared sensor (Nipro CE Thermo, NIPRO, Osaka, Japan) was used to measure ear canal temperature as an index of internal temperature. We recognized that this temperature has limitations as an index of internal temperature, but preferred to minimize any mental stresses associated with measurements. In our previous studies (Ogoh et al. 2013; Shibasaki et al. 2017), changes in temperature measured by the device were similar to those in esophageal temperature until temperature elevations up to 1.4°C with or without face cooling. Since ear canal temperature did not reach the upper limit, these data appear to be reliable.

### Perspective

The brain has the capacity for morphological and functional changes, and exercise training may elicit favorable changes in the structural and functional brain networks (Gomez‐Pinilla and Hillman 2013; Park and Friston 2013; Mandolesi et al. 2018). We previously found that cognitive processing was impaired at a given elevated internal temperature (Shibasaki et al. 2016, 2017). In the present study, we demonstrated that the effects of repetitive exercise on cognitive function were observed in the No‐go stimulus when internal temperature markedly increased, particularly in the hot condition. These results suggest that the neural activity of response inhibition (i.e., the amplitude of the No‐go‐P300 component) is more susceptible than that of response execution (i.e., the amplitude of the Go‐P300 component).

## Conflict of Interest

The authors declare no conflicts of interest. The results of the present study are presented clearly, honestly, and without fabrication, falsification, or inappropriate data manipulation. The results of this study do not constitute endorsement by the American College of Sports Medicine.
